# Knowledge and Clinical Approaches to Temporomandibular Disorders in Primary Healthcare: A Cross-Sectional Comparative Study of Physicians and Dentists in Croatia

**DOI:** 10.3390/clinpract16040070

**Published:** 2026-03-31

**Authors:** Dora Martic, Martin Miskovic, Antonija Palac Bzik, Ana Glavina, Ivan Kovacic, Antonija Tadin

**Affiliations:** 1 Department of Endodontics and Restorative Dental Medicine, Study of Dental Medicine, School of Medicine, University of Split, 21000 Split, Croatia; doramartic20@gmail.com (D.M.); martinmiskovic2001@gmail.com (M.M.); 2 Department of Prosthodontics, Dental Outpatient Clinic Zagreb, Perkovceva 3, 10000 Zagreb, Croatia; 3 Department of Dental Medicine University Hospital Center of Split, Spinciceva 1, 21000 Split, Croatia; glavina2014@gmail.com (A.G.); ivan.kovacic@mefst.hr (I.K.); 4 Department of Oral Medicine, Study of Dental Medicine, School of Medicine, University of Split, 21000 Split, Croatia; 5 Department of Prosthodontics, Study of Dental Medicine, School of Medicine, University of Split, 21000 Split, Croatia

**Keywords:** awareness, clinical practice, Croatia, dentists, knowledge, physicians, primary healthcare, temporomandibular disorders (TMDs)

## Abstract

**Objectives:** Temporomandibular disorders (TMDs) are common but often underrecognized and inadequately managed in primary healthcare, which may delay diagnosis and appropriate care. This study aimed to compare TMD-related knowledge, awareness, and clinical practices between dentists and physicians working in primary care and to identify factors associated with higher diagnostic confidence. **Methods:** A cross-sectional survey was conducted among dentists and physicians working in Croatian primary healthcare. TMD-related knowledge, clinical confidence, screening practices, and referral patterns were assessed using a structured questionnaire. **Results:** Dentists demonstrated significantly higher overall knowledge scores than physicians (15.6 ± 1.7 vs. 13.2 ± 4.1; *p* < 0.001), as well as greater diagnostic and therapeutic confidence (all *p* < 0.001). Routine TMD screening was reported by only 21.8% of participants, more frequently by dentists than physicians (36.1% vs. 8.2%; *p* < 0.001). Most respondents preferred referral rather than independent management. Regression analysis identified profession as the only independent predictor of higher TMD-related knowledge (*p* = 0.003). Insufficient knowledge, experience, and lack of confidence were the most reported barriers, particularly among physicians. **Conclusions:** The findings indicate clinically relevant gaps in TMD preparedness within primary healthcare, especially among physicians, despite frequent patient contact. Strengthening undergraduate and continuing education, promoting interdisciplinary training, and establishing clearer referral pathways may enhance early recognition and improve primary-level management of TMD.

## 1. Introduction

Temporomandibular disorders (TMDs) are defined as a diverse and heterogeneous set of neuromuscular and musculoskeletal conditions affecting structures of the temporomandibular joint and masticatory system. The clinical presentation of TMDs frequently involves pain, joint sounds such as clicking or crepitation, and limited mandibular movement, which may adversely influence mastication and overall oral function [1,2]. Current concepts describe TMDs as multifactorial conditions best explained by a biopsychosocial model, in which biological, behavioral, and psychological factors interact to influence symptom onset and persistence [2,3,4].

From an epidemiological perspective, temporomandibular disorders (TMDs) are among the most prevalent chronic orofacial pain conditions, with prevalence estimates spanning 5% to 30%, a variability that is largely attributable to differences in diagnostic criteria and population characteristics [5]. They are frequently associated with reduced quality of life, sleep disturbances, headache, and comorbid chronic pain conditions such as fibromyalgia, underscoring their broader relevance within chronic pain medicine [2,3,4].

Diagnosis of TMDs relies primarily on patient history and clinical examination. However, in primary healthcare settings, nonspecific and overlapping symptoms, frequent comorbidities, and limited formal training in orofacial pain substantially increase the risk of under-recognition, misdiagnosis, and delayed referral [6,7]. Diagnostic accuracy is strongly influenced by the clinician’s experience and familiarity with orofacial pain conditions [7]. Although TMDs are often detected by dental professionals, many patients initially seek care from physicians—particularly general practitioners, neurologists, or otolaryngologists—because of overlapping symptoms such as headache, ear pain, or neck pain [8,9,10,11,12]. Insufficient awareness and diagnostic uncertainty among physicians may contribute to unnecessary imaging, delayed diagnosis, or inadequate treatment [13,14,15]. Previous studies have consistently demonstrated lower levels of TMD-related knowledge and diagnostic confidence among physicians compared with dentists, largely attributed to differences in education and clinical exposure [13,14,15]. Given that effective management often requires collaboration between dental and medical professionals, improved interdisciplinary understanding and referral pathways are essential for patient-centered care [9,13,14].

Recent research across healthcare professions has further shown that gaps in knowledge, diagnostic confidence, and consistency of care persist among both dentists and physicians. These deficiencies are commonly linked to insufficient curricular coverage, limited interprofessional education, and restricted clinical exposure to TMDs, highlighting the need for coordinated, evidence-based educational strategies in primary healthcare [12,16,17,18,19,20,21,22,23,24].

In Croatia, available research reflects global trends but remains limited in scope. Studies conducted among students and adult populations have reported a high prevalence of TMD symptoms, often associated with perceived stress, sleep disturbances, and parafunctional behaviors [25,26,27,28]. Clinical and imaging-based studies have confirmed the heterogeneity of TMD presentations, including internal derangements and degenerative changes [27,28]. Additional findings suggest links between oral behaviors, psychological profiles, and genetic factors related to pain modulation, further supporting the multifactorial nature of TMDs [29,30,31,32]. Nevertheless, despite growing evidence, data on how well physicians and dentists in Croatian primary healthcare recognize and manage TMDs in everyday practice remain scarce. This gap is relevant because early identification and appropriate management of TMD at the primary-care level are important for timely intervention and efficient referral. Therefore, this study aimed to evaluate and compare TMD-related knowledge, awareness, and clinical practices among dentists and physicians working in Croatian primary healthcare. It was hypothesized that dentists would demonstrate higher levels of TMD-related knowledge and self-perceived clinical competence than physicians, reflecting differences in education and clinical exposure. The findings are intended to inform targeted educational interventions and support improved interdisciplinary management of TMD in primary care.

## 2. Materials and Methods

### 2.1. Ethics

Ethical approval for this study was granted by the Ethics Committee of the School of Medicine, University of Split (Class: 029-01/24-02/0001; Reg. No.: 2181-198-03-04-23-0028), within the framework of a larger study. The research was conducted in accordance with the Declaration of Helsinki and relevant national and institutional ethical standards. Study information was provided on the first page of the online survey, and informed consent was obtained implicitly, as continuation to the questionnaire indicated voluntary agreement to participate. Confidentiality and anonymity were strictly maintained throughout the study. Reporting transparency and methodological quality were ensured by adherence to the Checklist for Reporting Results of Internet E-Surveys (CHERRIES) (Supplemental File S1) [33].

### 2.2. Study Design, Participants and Data Collection

This cross-sectional online study was conducted from 1 September to 31 December 2024 at the Department of Restorative Dental Medicine and Endodontics, School of Medicine, University of Split. The target population comprised physicians (MD) and dentists (DMD) employed in Croatian primary healthcare. The survey link was distributed by e-mail to approximately 3000 professional addresses identified from institutional and professional medical websites. To improve response rates, the invitation was sent in three waves, each three weeks apart. The survey was hosted on Google Forms [34], and completion took approximately ten minutes. To minimize duplicate responses, the survey was configured to limit submissions to one response per Google account. No additional tracking measures (such as IP address monitoring or cookies) were implemented to preserve anonymity.

Eligible participants were physicians and dentists currently working in Croatian primary healthcare, fluent in Croatian, with at least one year of professional experience. Exclusion criteria were retirement, or employment outside Croatia. The study included only general dentists (without specialist training) and general or family medicine physicians practicing in primary healthcare. Healthcare professionals with any other medical or dental specialty were excluded to ensure a homogeneous primary care sample.

National data report a total of 16,107 physicians and 4013 dentists currently employed in Croatia [35]. Based on this population, a minimum sample size of 376 for MD and 351 for DMD was calculated using the Raosoft Sample Size Calculator, assuming a 95% confidence level and a 5% margin of error [36].

An invitation explaining study objectives, inclusion criteria, estimated completion time, and researcher contact details was distributed via e-mail. Recipients were encouraged to share the link with colleagues using a non-probability snowball sampling method. Participation was voluntary, with no incentives provided. Because the survey was disseminated through open distribution channels, the total number of individuals who received the invitation could not be detected, and therefore an exact response rate could not be calculated.

### 2.3. Questionnaire

The questionnaire was designed to assess knowledge, awareness, and clinical practices regarding TMDs, based on previous literature [14,15,16,17,18,19,20,21,22,23,24,37,38,39,40,41,42,43,44,45]. It was initially created by a sixth-year dental medicine student under supervision of the specialist in restorative dental medicine and endodontics and reviewed by a prosthodontics specialist for content validity, clinical relevance and clarity. A pilot test involving 30 healthcare professionals (15 physicians and 15 dentists) ensured comprehensibility and technical functionality. Minor wording changes were made following feedback, and pilot data were excluded from the final analysis.

The final version comprised 31 items (Supplemental File S2). Section S1 collected demographic and professional data (gender, age, education, workplace, years of clinical experience) (Q1–Q5). Section S2 assessed TMD knowledge with 17 items covering signs, symptoms, and risk factors (e.g., orofacial pain, joint sounds, limited mandibular movement, bruxism, psychological stress, trauma, dental procedures) (Q6–Q22). For each questionnaire item, respondents could choose one of three response options: “Yes,” “No,” or “I do not know.” Knowledge was assessed using a total score ranging from 0 to 17, where higher scores reflected a higher level of TMD-related knowledge. One point was awarded for each correct (“Yes”) response, whereas incorrect responses (“No” and “I do not know”) were scored as zero. Knowledge levels were subsequently classified according to Bloom’s taxonomy as good (80–100%; 14–17), moderate (60–79%; 10–13), or poor (<60%; 0–9) [46]. Clinical approaches to TMD were addressed in Section S3 through five items (Q23–Q27), and Section S4 measured self-rated knowledge and diagnostic confidence using a five-point Likert scale, subsequently condensed into three analytical categories. (Q28–Q31).

The questionnaire was administered as a single-page Google Forms survey comprising 31 items organized into thematic sections addressing demographic characteristics, TMD-related knowledge, clinical practices, and self-assessed confidence. All items were presented on a single screen without pagination. All questions were set as mandatory, and the survey could only be submitted after all items had been completed, ensuring complete datasets for analysis. Participants were able to review and modify their responses prior to final submission using the standard Google Forms review option.

### 2.4. Data Analysis

IBM SPSS Statistics (version 26.0; IBM Corp., Armonk, NY, USA) was used for all statistical analyses. Statistical significance was set at *p* < 0.05. Categorical variables were summarized as frequencies and percentages, whereas continuous variables were expressed as means and standard deviations. Normality of data distribution was assessed using the Shapiro–Wilk test. Group differences between physicians and dentists were examined using the chi-square test or Fisher’s exact test, as appropriate. Multiple linear regression analysis was subsequently applied to identify predictors of knowledge level, with regression coefficients (β) and 95% confidence intervals (CI) reported.

## 3. Results

### 3.1. Participant Demographic and Professional Characteristics

A total of 733 participants completed the survey (357 DMDs, 376 MDs). The mean age was 37.1 ± 11.1 years; dentists were younger than physicians (35.5 ± 9.5 vs. 38.6 ± 12.1 years). Most respondents were female (79.3%), with no gender difference between groups (Table 1).

**Table 1:** Demographic and Professional Characteristics of Respondents.

Characteristic/Question		Total *N* = 733	DMD *N* = 357	MD *N* = 376	*p*-Value
Gender	Male	152 (20.7%)	68 (19.0%)	84 (22.3%)	0.276
	Female	581 (79.3%)	289 (81.0%)	292 (77.7%)	
Age (years)	≤30	218 (29.7%)	100 (28.0%)	118 (31.4%)	0.012
	31–40	287 (39.2%)	170 (47.6%)	117 (31.1%)	
	41–50	115 (15.7%)	49 (13.7%)	66 (17.6%)	
	>51	113 (15.4%)	38 (10.6%)	75 (19.9%)	
Working Experience	≤5	300 (40.9%)	156 (43.7%)	144 (38.3%)	≤0.001
	6–10	159 (21.7%)	93 (21.6%)	66 (17.6%)	
	11–20	138 (18.8%)	59 (16.5%)	79 (21.0%)	
	>20	136 (18.6%)	49 (13.7%)	87 (23.1%)	
Type of Workplace	Private	111 (15.1%)	69 (19.3%)	42 (11.2%)	0.003
	Public	622 (84.9%)	288 (80.7%)	334 (88.8%)	

Abbreviations: DMD, Doctor of Dental Medicine; MD, Doctor of Medicine. Data are presented as frequencies (percentages). Chi-square test or Fisher exact test.

### 3.2. TMD Knowledge

The distribution of correct answers to questions assessing knowledge related to temporomandibular disorders (TMDs) is presented in Table 2. Overall, participants demonstrated a high level of awareness regarding TMD signs, symptoms, and etiological factors, with a mean knowledge score of 14.5 ± 3.2 out of 17. Dentists scored higher than physicians (15.6 ± 1.7 vs. 13.2 ± 4.1, *p* < 0.001), corresponding to “good” and “moderate” knowledge levels, respectively.

### 3.3. Clinical Management and Practice Patterns Regarding TMDs

As shown in Table 3, only 21.8% of respondents reported routinely examining patients for temporomandibular disorders (TMDs), with a significant difference between dentists and physicians (36.1% vs. 8.2%; *p* < 0.001). Although 60.8% agreed that TMD assessment should be part of standard evaluation, implementation in daily practice remained limited (*p* < 0.001). The most common barriers to independent management were insufficient experience or knowledge (39.0%) and lack of confidence (23.1%), both more frequent among physicians (*p* < 0.001).

### 3.4. Self-Assessment of Knowledge, Clinical Exposure, and Confidence in TMD Management

Self-assessment data (Table 4) showed marked interprofessional contrasts in exposure and confidence. Over half of physicians (54.3%) rarely encountered TMD patients, whereas most dentists (77.6%) reported occasional encounters (*p* < 0.001). Dentists displayed higher diagnostic (68.1% vs. 41.2%; *p* < 0.001) and therapeutic confidence (44.5% vs. 21.8%; *p* < 0.001). Regarding self-perceived knowledge, 54.6% of dentists and 20.2% of physicians rated their understanding as adequate (*p* < 0.001).

### 3.5. Factors Associated with TMD-Related Knowledge: Multiple Linear Regression Analysis

Multiple linear regression analysis (Table 5) showed that profession was the only variable significantly associated with higher TMD-related knowledge within the tested model (*p* = 0.003).

## 4. Discussion

In this cross-sectional analysis of Croatian primary healthcare professionals, dentists achieved higher TMD-related knowledge scores; however, physicians also displayed a moderate level of objectively assessed knowledge, indicating essential familiarity with the main signs, symptoms, and risk factors associated with TMDs. The observed differences may be associated with variations in undergraduate education, curricular focus, and routine clinical exposure to TMD, which are generally more emphasized in dental training than in medical education [37,38]. Similar patterns have been reported in previous studies, in which dental professionals demonstrated higher TMD-related knowledge compared with other non-dental healthcare professions [15,22,23,24]. Rather than reflecting inherent differences in professional competence, these findings suggest that training structure and clinical exposure may influence knowledge levels and clinical confidence [39,40,41]. From an educational and clinical perspective, the results highlight the potential value of strengthening and better aligning TMD-related educational content within medical and dental curricula to support more consistent recognition and management of TMD in primary healthcare. In line with these observations, one previous study has demonstrated considerable uncertainty among general medical practitioners regarding the diagnosis and management of temporomandibular disorders, with limited awareness of established guidelines and diagnostic tools, highlighting the need for improved education and clearer clinical pathways for early identification and referral of TMD patients [15].

In the present study, gender, age, and professional experience were not identified as significant predictors of knowledge related to temporomandibular disorders. In contrast, a study conducted among Italian dentists reported significant associations between knowledge levels and gender, age, and years of clinical experience, with knowledge decreasing as age and length of practice increased and higher knowledge scores observed among women [20]. Similarly, research conducted among dentists in Tehran found that increasing age and years of professional practice were associated with weaker attitudes and lower engagement toward TMD [42]. Lower TMD-related knowledge among older clinicians reported in some studies may reflect differences in educational background, as earlier training often preceded the introduction of standardized diagnostic criteria and contemporary TMD concepts. In addition, longer clinical experience may be associated with reliance on established practice patterns and less frequent engagement with updated guidelines unless supported by targeted continuing education [43].

Overall awareness of TMD manifestations was high, with recognition rates exceeding 70% for most signs, symptoms, and risk factors, including both musculoskeletal and psychosocial components, and was broadly comparable to previous international reports [18,20,41,44]. Although dentists demonstrated higher recognition rates for most TMD-related signs, symptoms, and risk factors, particularly those requiring clinical examination and dental-specific knowledge, physicians also showed high awareness of general symptoms such as headache and neck or shoulder pain, indicating overlapping but complementary diagnostic perspectives. Similar findings have been reported among dentists in Tehran, where greater knowledge of TMD signs and symptoms than of etiological factors was attributed to curricular emphasis on clinical recognition [42]. In this light, the present results may suggest that existing educational approaches place more focus on symptom identification than on comprehensive understanding of TMD etiology, indicating a potential need for more balanced educational content [45].

A dentist or physician is often the first to recognize symptoms of TMD, and effective interdisciplinary collaboration between these professionals is crucial for accurate diagnosis and timely referral to the appropriate specialist, ensuring prompt initiation of optimal treatment [15,16,22,47,48]. Interdisciplinary management is particularly important in chronic TMD cases, involving coordinated care among dentists, physicians, physiotherapists, psychologists, otorhinolaryngologists, and speech pathologists [47,48,49]. In our study, participants reported rarely or only occasionally encountering patients with TMD, with dentists encountering them more frequently than physicians. In contrast, a Polish study [17] found that 56% of dentists reported frequent contact with TMD patients, while 46% regularly diagnosed or treated such cases. Despite generally satisfactory theoretical knowledge in our study, only a minority of respondents routinely screened for TMD, with most preferring referral to specialists rather than independent management. Although many acknowledged that TMD evaluation should be part of routine examination, its implementation in daily practice remained low, highlighting a persistent gap between knowledge and behavior. This referral-dependent pattern, coupled with limited diagnostic confidence, has also been observed in international surveys among dentists and physicians [16,20,21].

Dentists were more likely to perform clinical screening and refer patients to prosthodontists or oral medicine specialists, while physicians predominantly referred to maxillofacial surgeons and otorhinolaryngologists, reflecting divergent patient pathways [16,20,21]. In the UK, 89% of physicians referred TMD patients to general dental practitioners, versus 11% to oral and maxillofacial surgery [15]. In Saudi Arabia, 59% of physical therapists had never evaluated or treated TMD, though 85% were aware they could provide care; referrals were most often directed to orthodontists (65%), specialized physical therapists and general dentists (39%), and oral surgeons (26%) [22]. Insufficient training and limited confidence were the main barriers to independent management, underscoring the need for structured postgraduate programs emphasizing conservative care, patient communication, and standardized diagnostic algorithms [16,20,21]. Limited TMD recognition has substantial public health and economic implications, as delayed diagnosis contributes to chronic pain, higher healthcare utilization, and reduced productivity, while early conservative management reduces long-term costs [5,20,21,22]. Strengthening primary-care diagnostic competence is therefore a cost-effective strategy for national healthcare planning.

The gap between self-perceived and objectively measured TMD knowledge was more pronounced among physicians than dentists, coinciding with lower patient exposure and reduced diagnostic and therapeutic confidence. In the UK, 88% of general medical practitioners reported low TMD knowledge [15], whereas in our study 33% of physicians and 9% of dentists reported limited knowledge. Among dentists in our cohort, 55% considered their knowledge adequate, consistent with findings from Poland [17].

In our cohort, approximately 40% of participants refrained from managing TMD due to insufficient experience or knowledge, and about 24% cited lack of confidence, with both barriers more pronounced among physicians than dentists. Similar patterns of underconfidence linked to educational gaps have been reported among other healthcare professionals involved in orofacial pain management, including physical therapists [24]. Likewise, an Indian study found that dentists frequently avoided treating TMD patients because of insufficient knowledge, low confidence, diagnostic complexity, prolonged treatment duration, and concerns about poor prognosis, whereas patient-related factors played a lesser role [50]. These findings support our regression analysis, in which profession emerged as the sole independent predictor of TMD-related knowledge, underscoring the central role of training structure and routine clinical exposure. Clinically, these findings suggest that limited confidence and knowledge in primary care may delay early recognition and conservative management of TMD, potentially prolonging patient symptoms and increasing reliance on specialist referral.

The findings of this study should be interpreted considering several methodological limitations. First, the cross-sectional design and reliance on a self-reported online questionnaire may have introduced information, recall, and social desirability bias, as participants’ responses reflect perceived rather than objectively measured knowledge and clinical performance. Although the use of closed-ended questions enabled standardized scoring and facilitated comparison between professional groups, it may not have fully captured clinical reasoning, decision-making processes, or real-world application of TMD-related knowledge. Second, voluntary participation combined with a non-probability snowball sampling strategy may have resulted in self-selection and selection bias, thereby limiting the representativeness of the sample. As the survey was disseminated through open distribution channels, the total number of individuals who received the invitation could not be determined, and an exact response rate could not be calculated. Consequently, the findings should be interpreted with caution and should not be generalized to all physicians and dentists working in Croatian primary healthcare. Although the sample size was substantial and participants were recruited from primary healthcare settings across Croatia, the gender distribution was uneven. While this likely reflects the feminization trend observed in healthcare professions, it may further affect generalizability. In addition, the study included only general dentists and general or family medicine physicians, whereas medical and dental specialists involved in TMD management were not assessed. This limits insight into interdisciplinary differences across levels of care and should be considered when interpreting referral patterns and management strategies. Another important limitation is that knowledge and diagnostic confidence were assessed exclusively through self-report rather than objective clinical evaluation. No direct observation of clinical performance, standardized case-based testing, or OSCE-based assessment was conducted, which may limit the accuracy of conclusions regarding actual clinical competence. Finally, the limited psychometric validation of the questionnaire and the scoring approach may have restricted its ability to capture more nuanced or in-depth understanding of TMD-related knowledge.

Despite the limitations, this study has notable strengths, as it is, to our knowledge, the first nationwide investigation examining differences in TMD-related knowledge, diagnostic confidence, and clinical practice patterns between physicians and dentists working in Croatian primary healthcare. The relatively large and geographically diverse sample enhances the relevance of the findings within the national context and provides an important baseline for future research and policy planning. Importantly, the results highlight specific, actionable gaps in early TMD recognition and management at the primary-care level, underscoring the need for the implementation of basic TMD screening protocols and clearer interprofessional referral pathways. Future studies should expand on these findings by including medical and dental specialists involved in TMD care to obtain a more comprehensive understanding of interdisciplinary competencies. In addition, the use of probability-based sampling, objective measures of clinical competence, and longitudinal designs would allow for stronger causal inference and evaluation of targeted educational and curricular interventions. Such approaches could inform structured orofacial pain education in medical curricula and support the development of standardized primary-care strategies for earlier diagnosis and more effective management of temporomandibular disorders.

## 5. Conclusions

Within the limitations of this study, the findings suggest differences in self-assessed TMD-related knowledge and clinical confidence between dentists and physicians in primary healthcare. Although dentists reported higher knowledge and confidence, these differences should be interpreted cautiously, and higher perceived knowledge did not consistently translate into routine screening or independent TMD management in either group. The overall low screening rates highlight potential gaps between education and clinical practice. These results support the need for improved interdisciplinary collaboration and more structured education on TMDs in primary care, while further research using representative samples and objective assessment methods is needed to inform educational and organizational strategies.

## Figures and Tables

**Table 2:** Distribution of Correct (“Yes”) Answers to Statements Evaluating Participants’ Knowledge and Awareness of Temporomandibular Disorders (TMDs).

Signs, Symptoms and Risk Factors Associated with TMD Pathology	Total *N* = 733	DMD *N* = 357	MD *N* = 376	*p*-Value
Headache	645 (88.0%)	333 (93.3%)	312 (83.0%)	≤0.001
Pain in Neck or Shoulder	517 (70.5%)	273 (76.5%)	244 (64.9%)	≤0.001
Orofacial Pain	697 (95.1%)	343 (96.1%)	354 (94.1%)	0.150
Limited Jaw Movement	723 (98.6%)	357 (100%)	366 (97.3%)	≤0.001
Ear Pain	681 (92.9%)	335 (93.8%)	346 (92.0%)	0.208
Difficulty Chewing	723 (98.6%)	357 (100%)	366 (97.3%)	≤0.001
Bruxism/Clenching	689 (94.0%)	354 (99.2%)	335 (89.1%)	≤0.001
TMJ Sounds During Movement	712 (97.1%)	352 (98.6%)	360 (95.7%)	0.017
Limited Mouth Opening or Mandibular Deviation	712 (97.1%)	356 (99.7%)	356 (94.7%)	≤0.001
Masseter Muscle Hypertrophy	584 (79.7%)	311 (87.1%)	273 (72.6%)	≤0.001
Tenderness of Masticatory Muscles	654 (89.2%)	335 (93.8%)	319 (84.8%)	≤0.001
TMJ Tenderness	714 (97.4%)	353 (98.9%)	361 (96.0%)	0.012
Occlusal Factors	556 (75.9%)	330 (92.4%)	226 (60.1%)	≤0.001
Facial Trauma	583 (79.5%)	295 (82.6%)	288 (76.6%)	0.026
Psychological Stress	596 (81.3%)	337 (94.4%)	259 (68.9%)	≤0.001
Orthodontic Therapy	522 (71.2%)	294 (82.4%)	228 (60.6%)	≤0.001
Recent Dental Procedure	485 (66.2%)	254 (71.1%)	231 (61.4%)	0.003

Abbreviations: DMD, Doctor of Dental Medicine; MD, Doctor of Medicine. Data are presented as frequencies (percentages). Chi-square test or Fisher exact test.

**Table 3:** Participants’ Clinical Approaches, Management Strategies, and Referral Patterns Regarding Temporomandibular Disorders (TMDs) and Orofacial Pain.

Characteristic/Question		Total *N* = 733	DMD *N* = 357	MD *N* = 376	*p*-Value
Regular TMD examination in clinical practice (“Yes”)		160 (21.8%)	129 (36.1%)	31 (8.2%)	≤0.001
Perceived importance of TMD assessment (“Yes”)		466 (60.8%)	270 (75.6%)	176 (46.8%)	≤0.001
Management of Patients with TMD	Independently	81 (11.1%)	38 (10.6%)	43 (11.4%)	0.412
	Refers to Specialist	652 (88.9%)	319 (89.4%)	333 (88.6%)	0.389
Referral of a Patient with TMD to a Specialist (Multiple Responses Possible)	MD	25 (3.4%)	12 (3.4%)	13 (3.5%)	0.553
	DMD	192 (26.2%)	25 (7.0%)	167 (44.4%)	≤0.001
	Maxillofacial Surgeon	296 (40.4%)	69 (19.3%)	227 (60.4%)	≤0.001
	Oral Surgeon	159 (21.7%)	79 (22.1%)	80 (21.3%)	0.424
	Oral Medicine Specialist	91 (12.4%)	31 (8.7%)	60 (16.0%)	0.002
	Prosthodontist	284 (38.7%)	267 (74.8%)	17 (4.5%)	≤0.001
	Otorhinolaryngologist	117 (16.0%)	21 (5.9%)	96 (25.5%)	≤0.001
	Neurologist	61 (8.3%)	37 (10.4%)	24 (6.4%)	0.034
	Orthodontist	71 (9.7%)	38 (10.6%)	33 (8.8%)	0.233
	Physiatrist	46 (6.3%)	23 (6.4%)	23 (6.1%)	0.488
	Other Specialists	42 (5.7%)	18 (5.0%)	24 (6.4%)	0.268
Reported Reasons for Not Providing Treatment (Multiple Responses Possible)	Insufficient Financial Compensation	17 (2.3%)	11 (3.1%)	6 (1.6%)	0.138
	Insufficient Experience or Knowledge	286 (39.0%)	109 (30.5%)	177 (47.1%)	≤0.001
	Lack of Confidence	169 (23.1%)	38 (10.6%)	131 (34.8%)	≤0.001
	Lack of Time	86 (11.7%)	40 (11.2%)	46 (12.2%)	0.376
	Other	338 (41.6%)	209 (58.5%)	129 (34.3%)	≤0.001

Abbreviations: DMD, Doctor of Dental Medicine; MD, Doctor of Medicine. Data are presented as frequencies (percentages). Chi-square test or Fisher exact test.

**Table 4:** Self-Assessment of Knowledge, Frequency of Patient Encounters, and Confidence in Diagnosing and Managing Patients with Temporomandibular Disorder (TMD).

Characteristic		Total *N* = 733	DMD *N* = 357	MD *N* = 376	*p*-Value
Frequency of Patient Visits with TMD	Rarely	284 (38.7%)	80 (22.4%)	204 (54.3%)	≤0.001
	Sometimes	449 (61.3%)	277 (77.6%)	172 (45.7%)	
	Often	0 (0%)	0 (0%)	0 (0%)	
Confidence in Diagnosing Patients with TMD	Unconfident	138 (18.8%)	30 (8.4%)	108 (28.7%)	≤0.001
	Neutral	197 (26.9%)	84 (23.5%)	113 (30.1%)	
	Confident	398 (54.3%)	243 (68.1%)	155 (41.2%)	
Confidence in Treating Patients with TMD	Unconfident	238 (32.5%)	88 (24.6%)	150 (39.9%)	≤0.001
	Neutral	254 (34.7%)	110 (30.8%)	144 (38.3%)	
	Confident	241 (32.9%)	159 (44.5%)	82 (21.8%)	
Self-Assessed Knowledge of TMD	Limited	153 (20.9%)	30 (8.4%)	123 (32.7%)	≤0.001
	Basic	309 (42.2%)	132 (37.0%)	177 (47.1%)	
	Adequate	271 (37.0%)	195 (54.6%)	76 (20.2%)	

Abbreviations: DMD, Doctor of Dental Medicine; MD, Doctor of Medicine. Data are presented as frequencies (percentages). Chi-square test or Fisher exact test.

**Table 5:** Association of Demographic and Professional Characteristics of Respondents Concerning the Combined Knowledge of Temporomandibular Disorders (TMDs).

Characteristic		TMD Knowledge β (95% CI)	*p*-Value
Gender	Man	−1.001 (−2.404–0.400)	0.161
	Woman	Reference	
Age	≤30	Reference	
	31–40	0.110 (−1.636–1.856)	0.902
	41–50	−1.364 (−4.185–1.485)	0.343
	≥51	1.796 (−5.995–0.403)	0.087
Profession	DMD	Reference	0.003
	MD	−2.150 (−3.583–−0.717)	
Working experience	≤5	Reference	
	6–10	0.337 (−1.549–2.223)	0.726
	11–20	0.859 (−0.852–2.570)	0.325
	≥20	−0.617 (−2.351–1.118)	0.468
Type of workplace	Private Practice	0.473 (−1.147–2.093)	0.567
	Health Center	Reference	
Frequency of Patient Visits with TMD	Rarely	Reference	
	Sometimes	−1.276 (−2.664–0.112)	0.072
Confidence in Diagnosing Patients with TMD	Unconfident	Reference	
	Neutral	1.183 (−1.504–3.870)	0.388
	Confident	2.342 (−0.374–5.059)	0.091
Confidence in Treating Patients with TMD	Unconfident	Reference	
	Neutral	−1.763 (−3.920–0.395)	0.109
	Confident	−1.061 (−3.370–1.248)	0.368
Self-Assessed Knowledge of TMD	Limited	Reference	
	Basic	1.332 (−0.973–3.637)	0.257
	Adequate	0.805 (−1.422–3.032)	0.479
Regular TMD examination in clinical practice (“Yes”)	Yes	1.068 (−0.338–2.474)	0.136
	No	Reference	
Perceived importance of TMD assessment (“Yes”)	Yes	0.050 (−1.265–1.365)	0.940
	No	Reference	
Management of patients with TMD	Independently	Reference	0.717
	Refers to Specialist	−0.242 (−1.552–1.067)	

Data are presented as β (95% CI). The reference knowledge category is “poor”. Abbreviations: β, β-regression coefficient, 95% CI, 95% confidence interval; DMD, Doctor of Dental Medicine; and MD, Doctor of Medicine.

## Data Availability

Data available on request due to privacy/ethical restrictions.

## Supplementary Materials

The following supporting information can be downloaded at: https://www.mdpi.com/article/10.3390/clinpract16040070/s1, File S1: CHERRIES Checklist for Web-based Survey; File S2: TMD_Questionnaire.
