# Supplementary material for: Knowledge and Clinical Approaches to Temporomandibular Disorders in Primary Healthcare: A Cross-Sectional Comparative Study of Physicians and Dentists in Croatia
Source: Clin Pract. 2026 Mar 31;16(4):70. doi: 10.3390/clinpract16040070 (PMC13114973; doi:10.3390/clinpract16040070)
Supplement: Supplementary file 1 [file clinpract-16-00070-s001.zip › TMD_Questionnaire.pdf]

File S2 TMD_Questionnaire

Section 1 – Demographic and Professional Characteristics of Respondents

1. Gender:
☐ Male ☐ Female

2. Age:

3. Profession:
☐ Doctor of Medicine (MD)
☐ Doctor of Dental Medicine (DMD)

4. Years of clinical professional experience:

5. Type of workplace:
☐ Public healthcare institution ☐ Private practice

Section 2 – Knowledge and Awareness of Temporomandibular Disorders (TMD)

Which of the following are signs, symptoms, and risk factors associated with temporomandibular disorder (TMD) pathology?

1. Headache	☐ Yes ☐ No ☐ I do not know
2. Pain in the neck or shoulder region	☐ Yes ☐ No ☐ I do not know
3. Orofacial pain	☐ Yes ☐ No ☐ I do not know
4. Limited jaw movement	☐ Yes ☐ No ☐ I do not know
5. Ear pain	☐ Yes ☐ No ☐ I do not know
6. Difficulty chewing	☐ Yes ☐ No ☐ I do not know
7. Bruxism or clenching	☐ Yes ☐ No ☐ I do not know
8. Temporomandibular joint sounds during jaw movement	☐ Yes ☐ No ☐ I do not know
9. Limited mouth opening or mandibular deviation	☐ Yes ☐ No ☐ I do not know
10. Masseter muscle hypertrophy	☐ Yes ☐ No ☐ I do not know
11. Tenderness of masticatory muscles	☐ Yes ☐ No ☐ I do not know
12. Tenderness of the temporomandibular joint	☐ Yes ☐ No ☐ I do not know
13. Occlusal factors	☐ Yes ☐ No ☐ I do not know
14. Facial trauma	☐ Yes ☐ No ☐ I do not know
15. Psychological stress	☐ Yes ☐ No ☐ I do not know
16. Orthodontic therapy	☐ Yes ☐ No ☐ I do not know

17. Recent dental procedures ☐ Yes ☐ No ☐ I do not know

Section 3 – Clinical Practice, Management, and Referral Patterns

1. Do you routinely examine patients for signs or symptoms of TMD: ☐ Yes ☐ No

2. Do you consider assessment of TMD important in routine examinations: ☐ Yes ☐ No

3. How do you usually manage patients with TMD: ☐ Independently ☐ Refer to specialist

4. If you refer patients, to which specialist(s)(Multiple answers possible):
☐ MD ☐ DMD ☐ Maxillofacial surgeon ☐ Oral surgeon

☐ Oral medicine specialist ☐ Prosthodontist ☐ ENT

☐ Neurologist ☐ Orthodontist ☐ Physiatrist ☐ Other

5. Reasons for not treating patients with TMD (Multiple answers possible):
☐ Insufficient financial compensation

☐ Insufficient experience or knowledge

☐ Lack of confidence

☐ Lack of time

☐ Other

Section 4 – Self-Assessment, Patient Encounters, and Confidence

1. Frequency of encounters with TMD patients:
☐ Never
☐ Rarely
☐ Sometimes
☐ Often
☐ Very often

2. Confidence in diagnosing TMD:
☐ Very unconfident
☐ Unconfident
☐ Neutral
☐ Confident
☐ Very confident

3. Confidence in treating TMD:
☐ Very unconfident
☐ Unconfident
☐ Neutral
☐ Confident
☐ Very confident

4. Self-assessed knowledge of TMD:
☐ Very limited
☐ Limited
☐ Moderate
☐ Good
☐ Very good
