# Supplementary material for: Knowledge and Clinical Approaches to Temporomandibular Disorders in Primary Healthcare: A Cross-Sectional Comparative Study of Physicians and Dentists in Croatia
Source: Clin Pract. 2026 Mar 31;16(4):70. doi: 10.3390/clinpract16040070 (PMC13114973; doi:10.3390/clinpract16040070)
Supplement: Supplementary file 1 [file clinpract-16-00070-s001.zip › CHERRIES Checklist_TMD.pdf]

Supplemental File S1. CHERRIES Checklist for Web-based Survey

Checklist for Reporting Results of Internet E-Surveys (CHERRIES)

Checklist Item	Explanation (as per CHERRIES)	Page Number
Describe survey design	Describe target population, sample frame. Is the sample a convenience sample? (In open surveys this is most likely.)	3-4
IRB approval	Mention whether the study has been approved by an IRB.	3-4
Informed consent	Describe the informed consent process, including survey length, data storage, investigator identity, and study purpose.	3-4
Data protection	If any personal information was collected or stored, describe mechanisms used to protect unauthorized access.	NA
Development and testing	State how the survey was developed and whether usability and technical functionality were tested before fielding.	3-4, 10-11
Open survey versus closed survey	Specify whether the survey was open to all visitors or restricted to a known sample.	3-4, 10-11
Contact mode	Indicate whether the initial contact with participants was made via the Internet.	3-4
Advertising the survey	Describe how and where the survey was announced or advertised, including wording of the announcement.	NA
Web/E-mail	State the type of e-survey and method used to capture responses.	3-4
Context	Describe the website or professional context in which the survey was	3-4

	distributed and potential influence on participation.	
Mandatory/voluntary	State whether participation was mandatory or voluntary.	3-4
Incentives	Report whether any incentives were offered.	3-4
Time/Date	Specify the timeframe of data collection.	3-4
Randomization of items	Describe whether questionnaire items were randomized.	NA
Adaptive questioning	Describe whether adaptive questioning was used.	NA
Number of items	State the number of questionnaire items per page.	3-4
Number of screens (pages)	State the number of pages/screens used.	3-4
Completeness check	Describe whether completeness or consistency checks were performed.	3-4
Review step	State whether respondents could review and change their answers.	3-4
Unique site visitor	Define how a unique visitor was identified.	NA
View rate	Report view rate if available.	NA
Participation rate	Report participation rate if available.	3-4, 10-11
Completion rate	Report completion rate if available.	3-4, 10-11
Cookies used	Indicate whether cookies were used to prevent duplicate entries.	3-4
IP check	Indicate whether IP address checks were used.	NA
Log file analysis	Describe whether log file analysis was used.	NA
Registration	Describe any registration or login requirement.	NA
Handling of incomplete questionnaires	Describe how incomplete questionnaires were handled.	3-4
Atypical timestamps	Describe whether submissions with atypical timestamps were excluded.	NA

Statistical correction	Describe any statistical correction applied.	NA

Modified from: Eysenbach G. Improving the quality of Web surveys: the Checklist for Reporting Results of Internet E-Surveys (CHERRIES). J Med Internet Res. 2004;6(3):e34.
